# *SmEIL1* transcription factor inhibits tanshinone accumulation in response to ethylene signaling in *Salvia miltiorrhiza*

**DOI:** 10.3389/fpls.2024.1356922

**Published:** 2024-04-02

**Authors:** Xiujuan Li, Man Xu, Ke Zhou, Siyu Hao, Liqin Li, Leran Wang, Wei Zhou, Guoyin Kai

**Affiliations:** ^1^ Zhejiang Provincial Traditional Chinese Medicine Key Laboratory of Chinese Medicine Resource Innovation and Transformation, Zhejiang Provincial International Science and Technology Cooperation Base for Active Ingredients of Medicinal and Edible Plants and Health, School of Pharmaceutical Sciences, Zhejiang Chinese Medical University, Hangzhou, China; ^2^ Dermatology department, Tianjin Academy of Traditional Chinese Medicine Affiliated Hospital, Tianjin, China; ^3^ Key Laboratory of Traditional Chinese Medicine for the Development and Clinical Transformation of Immunomodulatory Traditional Chinese Medicine in Zhejiang Province, Huzhou Central Hospital, The Fifth School of Clinical Medicine of Zhejiang Chinese Medical University, Hangzhou, China; ^4^ School of Life Sciences, Zhejiang Chinese Medical University, Hangzhou, China

**Keywords:** *Salvia miltiorrhiza*, ethylene, SmEIL1 transcription factor, tanshinone, regulatory mechanism

## Abstract

Among the bioactive compounds, lipid-soluble tanshinone is present in *Salvia miltiorrhiza*, a medicinal plant species. While it is known that ethephon has the ability to inhibit the tanshinones biosynthesis in the *S. miltiorrhiza* hairy root, however the underlying regulatory mechanism remains obscure. In this study, using the transcriptome dataset of the *S. miltiorrhiza* hairy root induced by ethephon, an ethylene-responsive transcriptional factor EIN3-like 1 (*SmEIL1*) was identified. The SmEIL1 protein was found to be localized in the nuclei, and confirmed by the transient transformation observed in tobacco leaves. The overexpression of *SmEIL1* was able to inhibit the tanshinones accumulation to a large degree, as well as down-regulate tanshinones biosynthetic genes including *SmGGPPS1, SmHMGR1, SmHMGS1, SmCPS1, SmKSL1* and *SmCYP76AH1*. These are well recognized participants in the tanshinones biosynthesis pathway. Further investigation on the *SmEIL1* was observed to inhibit the transcription of the *CPS1* gene by the Dual-Luciferase (Dual-LUC) and yeast one-hybrid (Y1H) assays. The data in this work will be of value regarding the involvement of *EILs* in regulating the biosynthesis of tanshinones and lay the foundation for the metabolic engineering of bioactive ingredients in *S. miltiorrhiza*.

## Introduction

*Salvia miltiorrhiza*, commonly known as Danshen, is a medicinal plant of the family *Lamiaceae* (Jia et al., 2019). Historically, the dried roots of Danshen were popular in the treatment of diseases related to the cardiovascular, digestive and cerebrovascular systems. They are also recorded as being able to perform pharmacological actions (Hu et al., 2014; Shi et al., 2019). Several Danshen products, such as the Danshen injection, continue to find wide usage in clinical practice. Given the wide application of *S. miltiorrhiza*, the study of medicinal compounds biosynthesis is important to ensure its production to meet clinical needs. From previous studies it appears that the principal active components of *S. miltiorrhiza* can be distinguished as either lipid-soluble or water-soluble components in nature (Liu et al., 2022). The lipid-soluble components include the tanshinones namely, tanshinone I (TA-I), tanshinone IIA (TA-II), dihydrotanshinone (DT), and cryptotanshinone (CT); the water-soluble components include the phenolic acids namely, salvianolic acid A (Sal A), salvianolic acid B (Sal B), caffeic acid (CA) and rosmarinic acid (RA) (Ma et al., 2015; Mao et al., 2020). Over the recent years, growing attention has been focused on the compounds above, mainly on ways to improve the tanshinones yield in *S. miltiorrhiza*. Tanshinone is confirmed to accumulate mainly in the *S. miltiorrhiza* roots, and three distinct stages can be observed in its biosynthesis pathway. First, the common terpenoid precursors, which include isopentenyl diphosphate (IPP) and dimethylallyl diphosphate (DMAPP) produced by two distinct processes, namely the mevalonate (MVA) pathway in cytosol and 2-C-Methyl-D-erythritol-4-phosphate (MEP) pathway in the plastids (Rohmer et al., 1993; Lange et al., 2000; Miziorko, 2011). Second, three known synthases are evident namely, copalyl diphosphate synthase 1 (CPS1), kaurene synthase-like 1 (KSL1), and miltiradiene oxidase (CYP76AH1); incidentally, several yet-to-be-identified enzymes are present, which form the skeleton of the tanshinones. Finally, through post-synthesis modifications namely, oxidation, methylation, decarboxylation, or cyclization, diverse tanshinones can be generated (Jiang et al., 2023). Data from several studies confirmed that the overexpression or downregulation of one or two synthase genes was able to promote the tanshinones biosynthesis process (Wang and Wu, 2010; Huang et al., 2019). The active ingredient present in medicinal plants can be enhanced by metabolic engineering strategy. This process requires a deep understanding of the biosynthesis pathway of the active ingredients, which includes the biosynthetic genes and the regulatory factors (Yu et al., 2020; Chen et al., 2022).

In plants, the transcription factors (TFs) were validated to regulate the processes of anti-stress and metabolic engineering (Zhou and Memelink, 2016; Sun et al., 2018; Yan et al., 2021). The EIN3/EIL1 is one member of the family of EIL transcription factors (Dolgikh et al., 2019), and it significantly affects plant growth and development, as well as the responses to biotic and abiotic stressors (Zhong et al., 2009; Zhu et al., 2022; Xu et al., 2023). The *Arabidopsis thaliana* genome has six EIN3/EIL1 members, while five are present in *Nicotiana tabacum*, six in *Oryza sativa* (rice), and six in *S. miltiorrhiza* (Solano et al., 1998; Zhang et al., 2018). Such types of TF possess many special structural features: 1. The N-terminal DNA binding domain has a highly conservative and unique folding structure. 2. The portion of the transmembrane amino acid shows acidic amino acid portions, five small basic amino acid clusters (BD I-V), and proline-abundant sections. 3. Coil structure (Wang et al., 2012). The coil structure is principally the region where the proteins interact and take part in DNA binding (Chao et al., 1997). In the EIL family, a rich alkaline amino acid is found to be close to the N-terminal helix motif of α spiral part, similar to the DNA binding domain observed in the bZIP family. The acidic amino acid portion, as well as the glutamine abundant and proline rich activation domains are regarded as the transcriptional activation domains (Kong et al., 2018). The presence of these motifs in the EIN3/EIL protein implies the possibility of their function in transcriptional regulation.

While ethylene is the simplest of the olefin gases, it is also the first gas molecule confirmed to have hormonal action and significantly promote the accumulation of the secondary metabolites in plants (Bleecker and Kende, 2000). In fact, EIN3/EIL1 is considered as one of the important regulatory factors, bearing a close relationship to ethylene signaling (Guo and Ecker, 2004). The EIN3 promotes the lengthening of the root hair through the direct activation of the RHD6-LIKE4 (RSL4) gene (Feng et al., 2017). Besides, the homeotic genes *CpEIN3a* and *CpNAC2* of the EIN3 family in papaya, influence the carotenoid biosynthesis by the direct transcription and activation of the expression of *CpPDS4* and *CpCHY-b* genes, which are linked to the biosynthesis of the carotenoid during the stage of ripening of the fruit (Fu et al., 2017). In apple, however, the homeotic gene *MdEIL1* of the EIN3 family directly binds to the *MdMYB1* promoter and transcriptionally activates its expression, showing an effect on the biosynthesis of anthocyanidin in apple fruit (An et al., 2018). However, the precise transcriptional mechanism in the ethylene-induced tanshinones biosynthesis continues to remain cryptic.

In this work, a new EIN3/EIL1 transcription factor *SmEIL1* was isolated and identified in *S. miltiorrhiza*. This study closely examined the role played by *SmEIL1* in the tanshinones biosynthesis induced by ethylene in *S. miltiorrhiza*, and gained insight into the regulatory network. Overall, our findings thus provide new insights into the underlying molecular mechanisms involved in the ethylene-induced tanshinones biosynthesis in *S. miltiorrhiza*.

## Materials and methods

### Plant materials and growth conditions

The *S. miltiorrhiza* plantlets were raised by adopting either the standard greenhouse conditions or the Murashige and Skoog (MS) plant growth medium. The temperature was maintained at 25°C, following a 16-h day and 8-h night cycle. The hairy roots of the *S. miltiorrhiza* were subcultured in 250 mL Erlenmeyer flasks, to which were added 100 mL 1/2 MS liquid medium. They were then placed in a shaker at 110 rpm/min in the dark at 25°C. The *Nicotiana benthamiana* were planted in the soil and raised in pots, maintaining the identical conditions as for the *S. miltiorrhiza*. Finally, RNA isolation was conducted using tissue samples drawn from the main root, lateral root, fibrous root, stem, petiole, young leaf, mature leaf, and flower tissues of a single one-year-old *S. miltiorrhiza* plant. Hairy roots of *S. miltiorrhiza* were expanded and cultured in 1/2 MS liquid medium with 70 μM ethephon treatment for about 50 days.

### Gene isolation and sequence analysis

Using the local datasets, identification of all the *EIL* families in *S. miltiorrihiza* was done. The *SmEIL1*, of the EIL family, was cloned by employing a homology-based cloning method, the details of which were explained earlier (Shi et al., 2016a). The primer pair utilized in the gene cloning was cited in the **Supplementary Table 1**. The BLAST-Protein (BLASTP) analysis of the *SmEIL1* was accomplished using the non-redundant (NR) protein sequence database (www.ncbi.nlm.nih.gov). Then four amino acid sequence highly homologous to SmEIL1 alignment was performed using DNAMAN software. Alignment sequences includes *Arabidopsis thaliana* AtEIL1 (accession number AT2G27050), *Arabidopsis thaliana* AtEIN3 (AT3G20770), *Lithospermum erythrorhizon* LeEIL1 (ACP56697.1) and *Malus domestica* MdEIL1 (ADE41153.1).Then, based on the amino acid sequences, a phylogenetic tree was built up using the neighbor-joining method and employing the MEGA 6.0 software. The bootstrap method was used to assess the reliability of each node in the tree and 1,000 replicates were done (Tamura et al., 2013).

### Subcellular localization analysis of *SmEIL1*

To verify the subcellular localization profile of *SmEIL1*, the open reading frame (ORF) of *SmEIL1* was cloned and constructed into the pHB-GFP vector driven by the CaMV35S promoter to form the *SmEIL1*-GFP fusion protein (**Supplementary Figure 5**). An empty pHB-GFP vector was used as the negative control. The fusion vector and the control vector were introduced into the *Agrobacterium rhizogenes* strain GV3101 for transient transformation, respectively. In order to observe the nuclei, a solution of 4’, 6-diamidino-2-phenylindole dihydrochloride (DAPI) at a concentration of 10 μg/mL was introduced into *N. benthamiana* leaves via syringe injection, 3-h of growth prior to microscopic examination. Then, the fluorescence signal was observed by laser confocal microscope (Zeiss, Germany) under the excitation of the 405 nm and 488 nm laser. The experiments were repeated with at least three biological replicates (Shi et al., 2016a).

### Gene expression profiles detected by quantitative real-time PCR

Quantitative real-time PCR (qRT-PCR) detection was done adopting the method used by Shi et al., 2016b. Different samples were collected from eight tissues including the main root, lateral root, fibrous root, stem, petiole, young leaf, mature leaf, and flower within a single one-year-old plant. Hairy roots treated with ethylene at 0, 1, 6, 12, and 24 h time points, were harvested and then frozen in liquid nitrogen. For the internal control, the *SmActin* gene was used. The primer sequences for the real-time PCR detection are listed in the **Supplementary Table 1**. Using the comparative *Ct* method, quantification of the gene expression levels was conducted (Shi et al., 2016b).

### Transformation of *SmEIL1* in *S. miltiorrhiza*

The open reading frame (ORF) of *SmEIL1* was placed in the double restriction insertion site of *Bam*HI and *Spe*I of the pHB vector and controlled by the CaMV35S promoter and *NOS* terminator (**Supplementary Figure 5**). To accomplish the *SmEIL1* knock-out (KO) vector construction, analysis of the potential gene editing sites of the *SmEIL1* gene sequence was done using the Optimized CRISPR Design (http://crispr.dbcls.jp/). Next, synthesis of a pair of complementary oligos was done. This was then ligated to the CRISPR/Cas9 system expression protein to enable the sgRNA and hSpCas9 to combine. Hence, the hSpCas9 was driven by the CaMV35S promoter, and the *SmEIL1* sgRNA was driven by the AtU6 promoter (**Supplementary Figure 6**). The expression cassette was then inserted into the linearized plant expression vector pCAMBIA1300 in order to infect the *S. miltiorrhiza* leaves and thus produce the hairy roots. For the control, the pCAMBIA1300 empty vector without the sgRNA sequence was used (**Supplementary Figure 6**). All the constructs were finally introduced into the *Agrobacterium* strain C58C1, which were then transformed into the *S. miltiorrhiza*, and the transgenic hairy root lines were generated as explained earlier on Huang et al., 2019.

### Detection of tanshinones by high-performance liquid chromatography

After continuous culture in 1/2 MS liquid medium for 60 days, each hairy root line was harvested and freeze dried for 24 h. Using high-performance liquid chromatography (HPLC), all the metabolites were quantified as described earlier (Huang et al., 2019; Sun et al., 2019; Huang et al., 2020). Next, the dried roots were finely powdered. Samples of 200 mg were then extracted with 16 mL of methanol/dichloromethane (3:1, v/v), sonicated for 1 h, and centrifuged at room temperature of 25°C. Post centrifugation, the supernatant was transferred into a distillation flask. The supernatant was centrifuged once more, poured into a distillation flask and spun dry at 50°C. Next, 2 mL of methanol was added into the distillation flask to dissolve the material present there. After one more centrifugation at 6,500 × g for 10 min, the samples were drawn out with a 1 mL sterile syringe and filtered separately through 0.22 µM organic membranes (Jinten, China). Finally, the resulting filtrate collected was prepared for HPLC detection (Huang et al., 2019).

### Dual-luciferase assay

Dual-LUC assays were done as cited earlier (Sun et al., 2019). The promoter portions of the candidate genes (~2000 bp) were inserted into a pGreen0800-LUC vector driving a firefly *LUC* reporter gene; the 35S promoter controlled the *Renilla luciferase* (REN) gene. Later, the recombinant vectors were transferred into *A. tumefaciens* (GV3101) along with a helper plasmid (pSoup-P19) encoding a cosuppressor. The test was performed as reported earlier (Sun et al., 2019). While the pHB-GFP construct was used as the negative control, and the *REN* gene was utilized as the internal control.

### Yeast one-hybrid assay

The yeast one-hybrid (Y1H) assay was done adopting the technique reported by Sun et al (Sun et al., 2019). The full-length coding sequence of the *SmEIL1* gene was constructed in combination with the pB42AD vector. Two EBS-box sequences in the promoter (5’-ATGAATCCT-3’ and 5’-TCCATGCA-3’) were inserted separately into the pLacZ2u plasmid in triplicate. The recombinants produced through this procedure were co-transformed into the EGY48a yeast strain. The transformants were cultivated on the SD/-Ura/-Trp medium for 48 h, and the positive binding activity was evident as a blue color on the SD/-Ura/-Trp medium with 5-bromo-4-chloro-3-indolyl-b-D-galactopyranoside (X-Gal) for 24 h. The empty vectors of pB42CE and pLacZ2u functioned as the negative controls.

### Statistical analysis

Each of the findings indicate the average value of three independently conducted experiments. In this study, all the experiments were performed in triplicate. The contents and degrees of gene expression involved in the biosynthesis of tanshinones and phenolic acids are given as the mean value ± SD. SPSS 16.0 software was employed to conduct the single-sample *t*-test and One-Way ANOVA; the *P*-value < 0.05 were regarded as statistically significant.

## Results

### Ethylene signaling inhibits the accumulation of tanshinones in the hairy roots of *S. miltiorrhiza*

**Figure 1:**
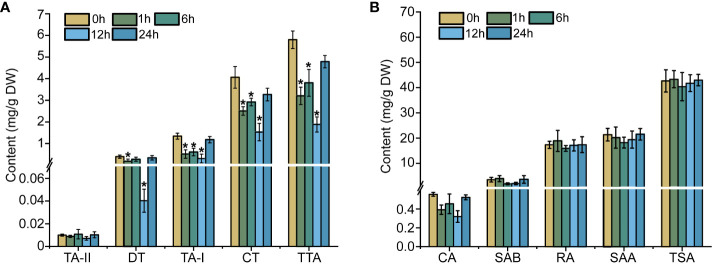
Effects of ethylene on tanshinones **(A)** and phenolic acids **(B)** in hairy roots. The error line indicates the error for three biological replicates; *t*-test was done for significant differences (*P< 0.05).

Ethylene is a gaseous hormone which exerts a significant effect on the plant in terms of growth, development, and stress responses (Bleecker and Kende, 2000). Initially, HPLC detection was used to examine the content of tanshinones and phenolic acid in the *S. miltiorrhiza* hairy roots treated with ethephon. Within 24-h of treatment, the total tanshinones content present in the *S. miltiorrhiza* hairy roots revealed a remarkable drop in comparison to the control group (0-h of treatment). All four tanshinone compounds achieved their lowest levels, post treatment of 12 hours duration. The dihydrotanshinone (DT) content particularly registered a 90% drop when compared to the control group, while the tanshinone I (TA-I) content exhibited a 4.4-fold reduction, and the cryptotanshinone (CT) level dropped by 77% in comparison to the control (**Figure 1A**). However, with the ethylene treatment from 1- to 24-h, the accumulation of phenolic acids did not reveal a very regulative variation (**Figure 1B**). The results cited above reiterate that ethylene signaling can inhibit the accumulation of tanshinones in the *S. miltiorrhiza* hairy roots.

### Ethylene signaling induced the expression of the key genes involved in the biosynthesis pathway of tanshinones

**Figure 2:**
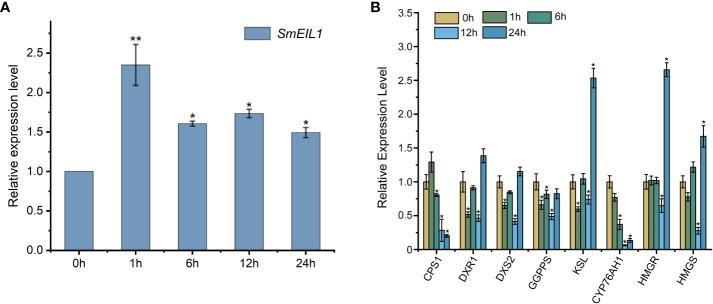
Expression profiles of *SmEIL1* gene and the genes involved in the tanshinones biosynthesis pathway in response to the treatment with ethylene. **(A)** The expression profiles of *SmEIL1* gene after the induction of ethylene. **(B)** Expression profiles of genes in the tanshinones biosynthesis pathway after treatment with ethylene. Fold changes of the relative gene expression level were all normalized to the 0 h treatment. The error line indicates the error for three biological replicates; *t*-test was done for significant differences (**P< 0.01; *P< 0.05).

To investigate whether the *SmEIL1* was induced by ethylene, the *SmEIL1* expression post the exogenous ethephon treatment in the *S. miltiorrhiza* hairy roots was examined by qRT-PCR at different time intervals from 0- to 24-h. The ethephon was then found to significantly upregulate the expression of *SmEIL1*, and its transcript level rapidly peaked after the 1 h ethephon treatment (**Figure 2A**). The expression levels of the principal genes that participated in the biosynthesis pathway of the tanshinones in the hairy roots after ethephon treatment were quantified using qRT-PCR detection. The eight genes involved in the tanshinones biosynthesis including copalyl diphosphate synthase 1 (*CPS1*), 1-deoxy-D-xylulose 5-phosphate reductoisomerase (*DXR1*), 1-deoxy-D-xylulose 5-phosphate synthase 1 (*DXS2*), geranylgeranyl pyrophosphate synthase (*GGPPS*), kaurene synthase-like 1 (*KSL*), miltiradiene oxidase (*CYP76AH1*), 3-hydroxy-3-methylglutaryl-coenzyme A reductase (*HMGR*) and 3-hydroxy-3-methylglutaryl coenzyme A synthase (*HMGS*) genes, exhibited visible variations with the treatment of ethephon. Of these, the *CPS1*, *GGPPS*, and *CYP76AH1* genes showed a maximum of 4-, 5.3-, and 3-fold reductions, respectively, when compared with the control (**Figure 2B**). This finding suggests that ethylene signaling can inhibit the expression of the three pivotal genes (*CPS1*, *GGPPS*, and *CYP76AH1*) involved in the tanshinones biosynthesis process.

### Isolation and sequence analysis of *SmEIL1* gene

**Figure 3:**
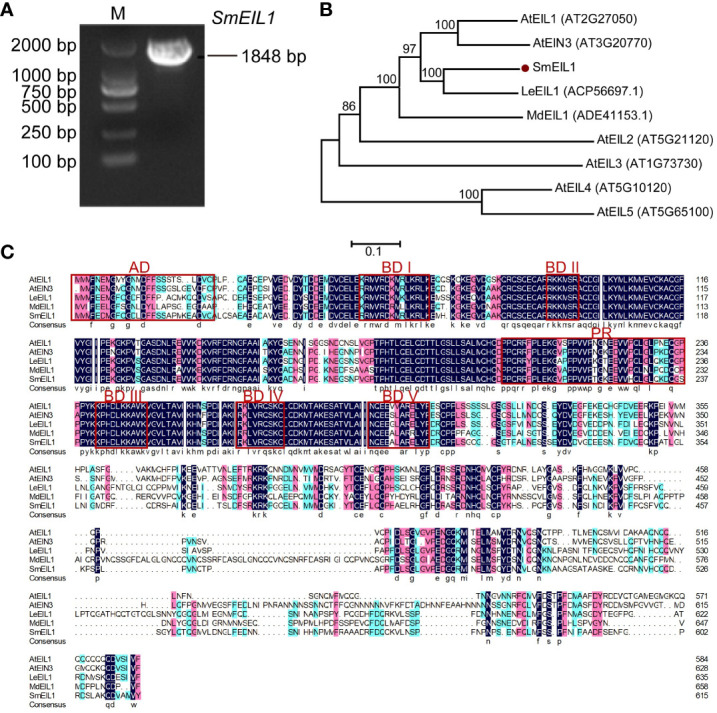
Characterization and subcellular localization of *SmEIL1*. **(A)** Cloning of *SmEIL1* gene. **(B)** Phylogenetic tree used in analyzing SmEIL1 and other EIL protein members in plants by neighbor-joining method, with MEGA 6 software. The numbers on the nodes indicate the bootstrap values post 1000 replicates. **(C)** Multiple protein sequences alignment between SmEIL1 and AtEILs transcription factors (AD, acidic amino acid regions; PR, Proline-rich regions; BD I-V, Basic amino acid regions I-V). The frame represents conserved domains.

It is a known fact that the *EIL1* gene is assumed to be the core regulator in ethylene signaling (Meng et al., 2018, 2018). In the study cited above, ethylene signaling has been verified as an inhibitor of tanshinones accumulation in the *S. miltiorrhiza* hairy roots. Therefore, cloning the *EIL1* gene and studying its molecular mechanism as a regulator in the biosynthesis of tanshinones is a worthwhile endeavor. Using the data of the *S. miltiorrhiza* transcriptome, the entire cDNA sequence of the *SmEIL1* gene in *S. miltiorrhiza* was firstly spliced, using the sequence of the *AtEIL1* (AT2G27050) gene in *Arabidopsis* as the reference. Soon after, the *SmEIL1* gene was isolated via homology-based cloning; the gene was found to be 1848-bp long and encoded 616 amino acids (**Figure 3A**). The *SmEIL1* protein is composed of several conserved motifs, such as the acidic amino acid portion (AD), alkaline amino acid part (BDI-V), and proline abundant (PR) domains (**Figure 3C**). The EIN3/EIL1 members of the family *Arabidopsis*, *Lithospermum erythrorhizon* LeEIL1, *Malus domestica* MdEIL1 and SmEIL1 were used to construct a phylogenetic tree. From the results, it was evident the closest evolutionary relationship existed between *SmEIL1* and LeEIL1 (**Figure 3B**). LeEIL1 acts as a positive regulator of purslane synthesis in the hairy roots of comfrey *Lithospermum erythrorhizon* and promotes purslane accumulation (Fang et al., 2016). Hence, this suggests that the *SmEIL1* gene plays a similar role in *S. miltiorrhiza*.

### Tissue expression and subcellular location analysis of *SmEIL1*

**Figure 4:**
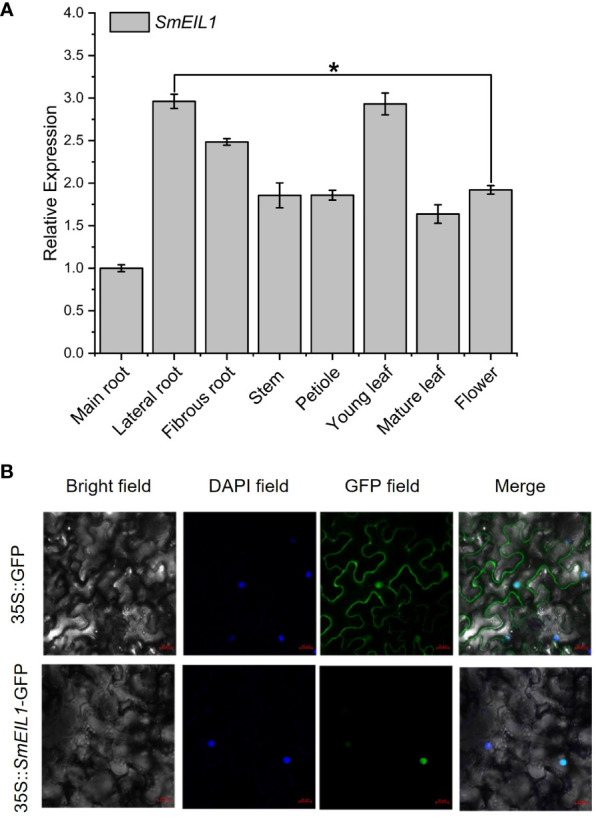
Tissue expression and subcellular location analysis of *SmEIL1*. **(A)** Expression patterns of the *SmEIL1* gene in different tissues. Fold changes in the relative gene expression level of all the other tissues are normalized to the main root. **(B)** Subcellular localization of *SmEIL1* in leaf epidermal cells of *N. benthamiana*. DAPI was the positive control. (The error line indicates the error for three biological replicates.) (*P<0.05).

An examination of the *SmEIL1* gene expression profiles in different tissues was conducted using qRT-PCR detection. The branch roots and young leaves exhibited the highest degree of expression, while the main roots showed the lowest degree of expression (**Figure 4A**). Later, the subcellular localization of the SmEIL1 was investigated in the tobacco cells via the transient expression of SmEIL1 combined with GFP. Vigorous fluorescence was evident only in the nuclei containing the construct of the 35S::*SmEIL1*-GFP recombinant; the control recombinant of 35S::GFP, however, displayed fluorescence throughout the whole cell (**Figure 4B**), which implies that the SmEIL1 protein is localized in the nuclei in *S. miltiorrhiza*.

### *SmEIL1* inhibits tanshinones biosynthesis in the transgenic hairy root of *S. miltiorrhiza*

**Figure 5:**
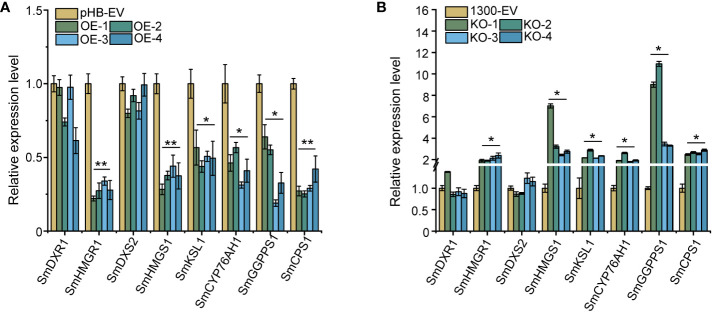
Gene expression levels of the main enzyme genes in tanshinones biosynthesis pathway in overexpression **(A)** and knock-out **(B)** hairy roots. The error line indicates the error for three biological replicates; t-test was done for significant differences (**P< 0.01; *P< 0.05).

On further investigation of whether *SmEIL1* can influence the tanshinones biosynthesis in the *S. miltiorrhiza* hairy roots, an examination was done of the over-expression (OE) and knock-out (KO) of the *SmEIL1* gene in the transgenic hairy root lines, independent of each other, through genomic PCR detection (**Supplementary Figure 1**); after this, DNA sequencing was introduced to select the positive hairy root lines (**Supplementary Figure 3**) and qRT-PCR assays (**Supplementary Figures 3**, **4**) were done as well. For further analysis, a selection was made of four independent OE lines (OE-1, OE-2, OE-3, and OE-4), which showed the greatest levels of expression, and four KO lines (KO-1, KO-2, KO-3, and KO-4) with the lowest expression levels. On comparison with the control lines, the four *SmEIL1* OE lines exhibited higher *SmEIL1* transcript levels (30 to 70-fold rise) (**Supplementary Figure 3**). Besides, the *SmHMGR1, SmHMGS1, SmGGPPS1, SmCPS1, SmKSL1* and *SmCYP76AH1* were examined to be remarkably downregulated in the four OE lines (**Figure 5A**). On the contrary, *SmHMGR1, SmHMGS1, SmCPS1* and *SmGGPPS1* were found to show a significant upregulation in all the four KO lines (**Figure 5B**). From these findings it become clear that the *SmEIL1* may act as a negative regulatory factor to participate in the tanshinones biosynthesis process.

**Figure 6:**
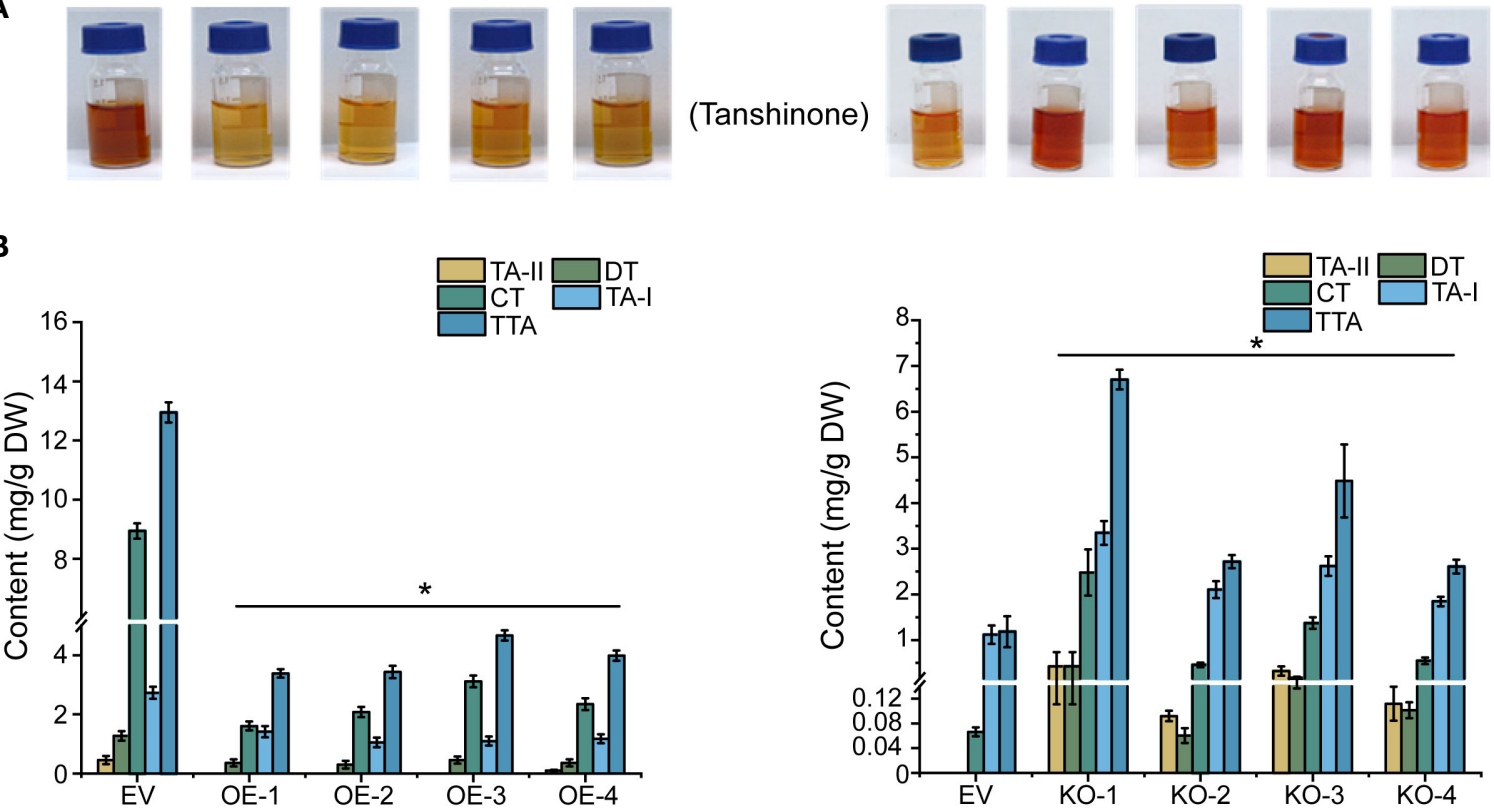
Extraction and determination of tanshinones in transgenic hairy roots. **(A)** Tanshinones extract from overexpression and knock-out hairy roots. **(B)** Determination of tanshinones content in overexpressed and knock-out hairy roots. The error line indicates the error for three biological replicates; *t*-test was done for significant differences (*P< 0.05).

In line with the gene expression profiles cited above, four tanshinones in the hairy root lines of 45-day-old, namely tanshinone I (TA-I), tanshinone II (TA-II), cryptotanshinone (CT), dihydrotanshinone (DT) and total tanshinones (TTA) were identified through HPLC detection. The TTA contents all exhibited a significant reduction in the four OE lines (OE-1, OE-2, OE-3, and OE-4); of these, the OE-1 line showed the lowest TTA concentration, dropping to 74% less than the control. Consistently, the TTA content in the four KO lines (KO-1, KO-2, KO-3, and KO-4) all showed significant elevation in the range from 2- to 4.66 times more than the control (**Figure 6**).

### *SmEIL1* inhibits the transcription of *SmCPS1* gene

**Figure 7:**
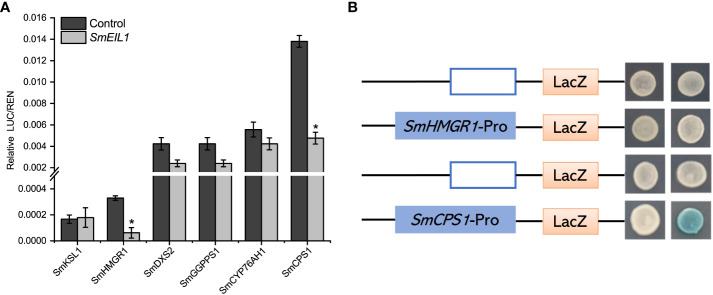
*SmEIL1* inhibits the *SmCPS1* expression. **(A)** Dual-Luc results of *SmEIL1* on key enzyme promoters in tanshinones synthesis pathway. The error line indicates the error for three biological replicates; *t*-test was done for significant differences (*P< 0.05). **(B)**Y1H assay. (AD: pB42AD vector.).

Dual-Luc and Y1H assays were performed to clarify the potential molecular mechanism controlled by *SmEIL1*, and confirmed the downstream target genes which participated in the tanshinones biosynthesis pathway. The Dual-Luc assay confirm that *SmEIL1* inhibits the biosynthesis of tanshinones, most probably by the inhibition of the transcription of the *SmHMGR1* and *SmCPS1* genes (**Figure 7A**). The specific binding element (EBS-box, A (C/T) G (A/T) A (C/T) CT) for the SmEIL1 protein is present in the promoter portion of the *SmHMGR1* and *SmCPS1* genes. The Y1H assay reveal that the SmEIL1 is unable to bind with the EBS-box element in the promoter region of the *SmHMGR1* gene; however, it can bind with the EBS-box element in the promoter portion of the *SmCPS1* gene. From these results it becomes clear that the *SmCPS1* may be a direct target for the *SmEIL1* to regulate the tanshinones biosynthesis in a negative manner.

## Discussion

Ethylene (ET) is a crucial phytohormone which modulates the plant secondary metabolites, growth, and adaptation to the environment (Heredia and Cisneros-Zevallos, 2009; Chen et al., 2012; Cheng et al., 2018; Ke et al., 2018; Li et al., 2018). In the signaling pathway of plant, *EIN3/EIL1* gene was thought to act as a core integration node between ET and other signals (Shi et al., 2012; Liang et al., 2013; Liu et al., 2017; Bai et al., 2020). With the mediation of *EIN3/EIL1* gene, ethylene can promote the accumulation of phenolic antioxidants in *Daucus carota* (Heredia and Cisneros-Zevallos, 2009), the production of shikonin and its derivatives in *Lithospermum erythrorhizon* (Fang et al., 2016),anthocyanin biosynthesis in apple (An et al., 2018), and the biosynthesis of capsaicinoids in *Capsicum* (Wen et al., 2022). ET-stabilized transcription factors EIN3 and EIL1 were validated to interact with multiple transcription factors to synergistically regulate anthocyanin accumulation, trichome formation, and defense against insect attack (Song et al., 2022). In the present work, with the treatment of ethephon in *S. miltiorrhiza* hairy roots, we revealed that the production of tanshinones including DT, CT, TA-I, and TTA were rapidly decreased at the induction time point of 1 h. Although the contents of DT, TA-I, CT and TTA recovered after 24 h of treatment, their contents were still lower than the control. This phenomenon may be caused by the fact that *S. miltiorrhiza* hairy roots in response to ET is usually short-term and rapid (Li et al., 2024). No visible variations were noted for the CA and SAB accumulation; however, the trend of variations in the RA, SAA and TSA biosynthesis showed irregularities (**Figure 1**). Hence, it pushes us to explore the molecular mechanism of how SmEIL1 mediate the ET signaling to regulate the biosynthesis of tanshinones in *S. miltiorrhiza*.

Based on the *S. miltiorrhiza* transcriptome collected in this study, the whole ORF sequence of *SmEIL1* was firstly assembled using the *AtEIL1* gene in *A. thaliana* as the reference sequence, and then it was cloned. Six members of *EIL* families were found in the *S. miltiorrhiza* transcriptome. In fact, six members are present in *A. thaliana* (Chao et al., 1997), four members in *S. lycopersicum* (Tieman et al., 2001; Yokotani et al., 2003), and five members in *N. tabacum* (Kosugi and Ohashi, 2000; Rieu et al., 2003). Obviously, *EIL* is a gene family and may conduct multiple functions by different gene members. In *A. thaliana*, the *EIN3*, *EIL1* and *EIL2* genes were observed to play a part in the ethylene signaling to regulate plant growth (Houben et al., 2022). The *LeEIL1* gene, in *Lithospermum erythrorhizon*, was validated to participate in regulating the biosynthesis of shikonin, whereas *MdEIL1* gene in *Malus domestica* was identified to promote the accumulation of anthocyanin (An et al., 2018). Through the construction of evolutionary tree, the SmEIL1 protein was found to fall into the same subgroup along with LeEIL1, implying that the *SmEIL1* might exert a regulatory influence on the secondary metabolites triggered by the ethylene signal in *S. miltiorrhiza*.

Through examining the expression profile of *SmEIL1* gene in diverse tissues, it was found that the lateral and fibrous roots, and young leaf showed a degree of expression much higher than the other tissues like those of the flower, mature leaf, petiole, stem, and main root (**Figure 4A**). While the leaf is an important vegetative organ which is able to receive the ethylene signals directly, the lateral and fibrous roots are thought to be the principal tissues for the accumulation of medicinal substances in *S. miltiorrhiza* (Zhou et al., 2021). Moreover, the relative expression of *SmEIL1* increased significantly in hairy roots treated with ethylene, reaching a peak after 1 h of treatment (**Figure 2A**). The tissue expression and induced expression profile of *SmEIL1* gene is similar to the *SmERF1b-like* gene, which shows a significant response to exogenous ethylene supply and exhibits the highest expression level in leaves after 1 h of induction in *S. miltiorrhiza* (Li et al., 2024). Soon after, the gain- and loss-of-function assays revealed that the *SmEIL1* was able to negatively regulate the ethylene-mediated accumulation of tanshinones in the *S. miltiorrhiza* hairy root lines (**Figures 5**, **6**). In fact, the total tanshinones concentration in the *SmEIL1* knock-out transgenic hairy root line was at maximum 4.66 times as that of the non-transgenic line (**Figure 5B**). The EBS-box (A (C/T) G (A/T) A (C/T) CT) element is considered essential in the binding by the EIL1 protein (Yamasaki et al., 2005), found in the promoter sequence of the *SmKSL1, SmHMGR1, SmDXS2, SmGGPPS1, SmCYP76AH1*, and *SmCPS1* genes. Later, the Dual-LUC and Y1H assays performed revealed that the *SmEIL1* directly activated its expression of the *SmCPS1* gene (**Figure 7**). The qRT-PCR assay revealed that the overexpression of *SmEIL1* in the four transgenic hairy roots not only inhibited the transcript level of its target gene *SmCPS1*, but also decreased the expression of three genes in the tanshinones biosynthesis pathway (**Figure 5**). The overexpression of the *SmERF1L1* encourages the expression of the *DXS2, DXR, HMGS, CPS1, and KSL1*; however, the *DXR* alone is the target of the SmERF1L1 (Huang et al., 2019). In the transgenic hairy roots, the overexpression of the *SmMYB1* activated the expression of the *PAL1*, *C4H1*, *4CL1*, *TAT1*, *HPPR1*, and *RAS1* genes; however, the *CYP98A14* alone was confirmed as the target of the SmMYB1 (Zhou et al., 2021). From these findings, it seems likely that the ectopic expression of certain transcriptional genes can regulate the degree of expression of their target genes as well as that of the other genes present in the same biosynthesis pathway.

**Figure 8:**
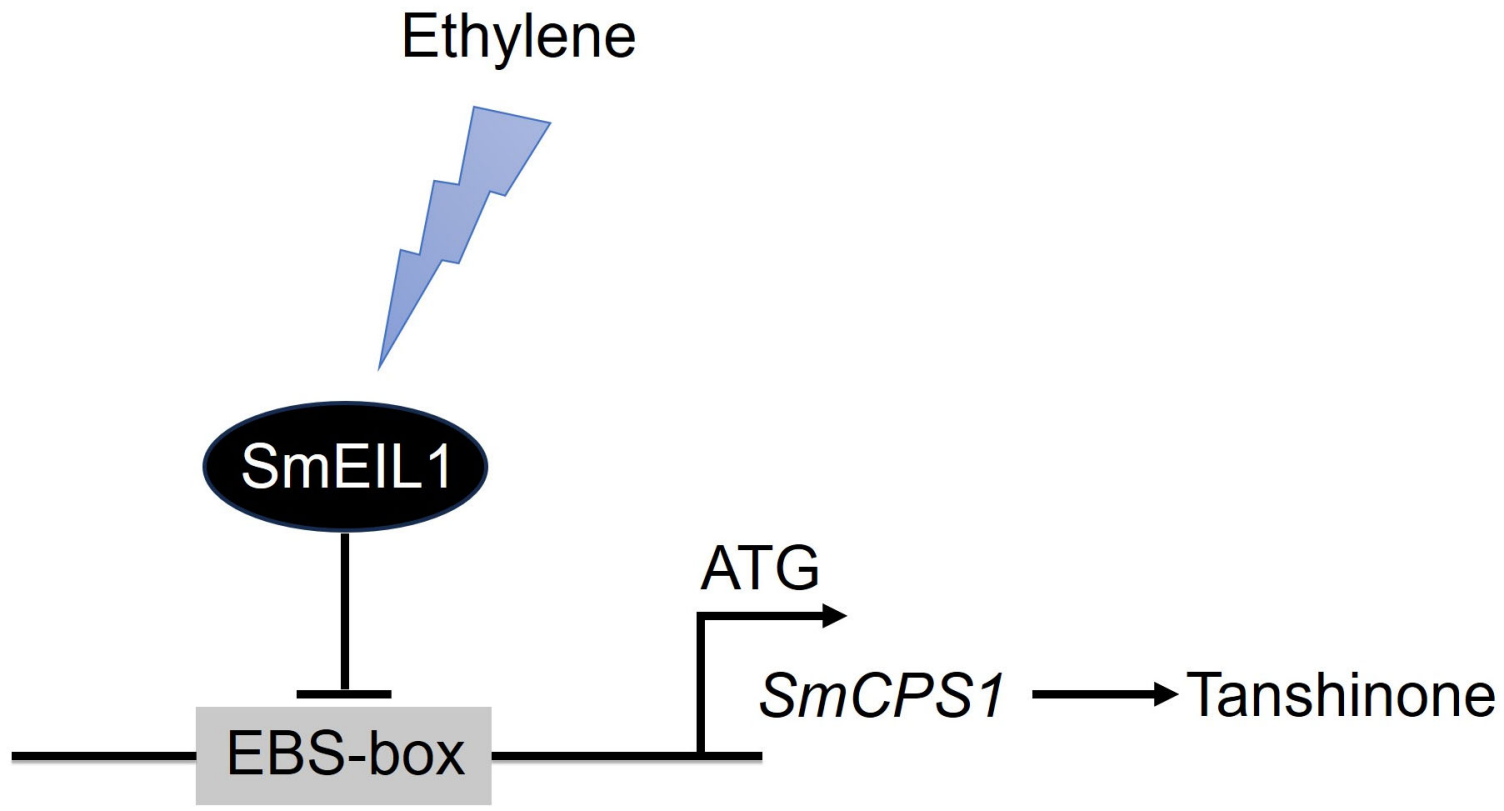
A model proposed for the role of *SmEIL1* in modulating tanshinones biosynthesis in *S. miltiorrhiza*.

The present work, to the best of our knowledge, is the first report on the identification of the tanshinones biosynthesis gene, acting as the direct target of the SmEIL1 protein, the ethylene signaling gene in *S. miltiorrhiza*. On considering the observations cited above, a model was proposed for the role of SmEIL1 as a negative regulator inhibiting tanshinones accumulation (**Figure 8**). On being exposed to triggering by the ethylene, the *SmEIL1* downregulates the transcription of the *CPS1* gene by binding with the EBS-box elements present in the promoter of the *CPS1* gene. This caused an inhibition of the tanshinones accumulation in the *S. miltiorrhiza* hairy roots (**Figure 8**).

## Data availability statement

The original contributions presented in the study are included in the article/**Supplementary Material**. Further inquiries can be directed to the corresponding authors.

## Author contributions

XL: Writing – original draft, Writing – review & editing. MX: Writing – original draft, Writing – review & editing. KZ: Writing – original draft. SH: Writing – original draft. LL: Writing – review & editing. LW: Writing – original draft. WZ: Writing – original draft, Writing – review & editing. GK: Writing – original draft, Writing – review & editing.
