# Supplementary material for: SmEIL1 transcription factor inhibits tanshinone accumulation in response to ethylene signaling in Salvia miltiorrhiza
Source: Front Plant Sci. 2024 Apr 2;15:1356922. doi: 10.3389/fpls.2024.1356922 (PMC11018959; doi:10.3389/fpls.2024.1356922)
Supplement: Supplementary file 1 [file DataSheet_1.docx]

Supplementary Material

***SmEIL1* Transcription Factor Inhibits Tanshinone Accumulation in Response to** **Ethylene Signaling in *Salvia miltiorrhiza***

**Xiujuan Li ^1†^, Man Xu ^1†^, Ke Zhou ^2†^, Siyu Hao ^1^, Liqin Li^3^, Leran Wang^4^,** **Wei Zhou ^1^*, Guoyin Kai ^1^***

†These authors contributed equally to this work and share first authorship

^1^Zhejiang Key TCM Laboratory for Chinese Resource Innovation and Transformation, School of Pharmaceutical Sciences, Zhejiang Provincial International S&T Cooperation Base for Active Ingredients of Medicinal and Edible Plants and Health, School of Pharmaceutical Sciences, Zhejiang Chinese Medical University, Hangzhou, China

^2^Dermatology department, Tianjin Academy of Traditional Chinese Medicine Affiliated Hospital

^3^Key Laboratory of Traditional Chinese Medicine for the Development and Clinical Transformation of Immunomodulatory Traditional Chinese Medicine in Zhejiang Province, Huzhou Central Hospital, the Fifth School of Clinical Medicine of Zhejiang Chinese Medical University

^4^School of Life Sciences, Zhejiang Chinese Medical University, Hangzhou, China

*** Correspondence:**

Wei Zhou
[zhouwei19810501@163.com](mailto:zhouwei19810501@163.com)

Guoyin Kai
[guoyinkai1@126.com](mailto:guoyinkai1@126.com)


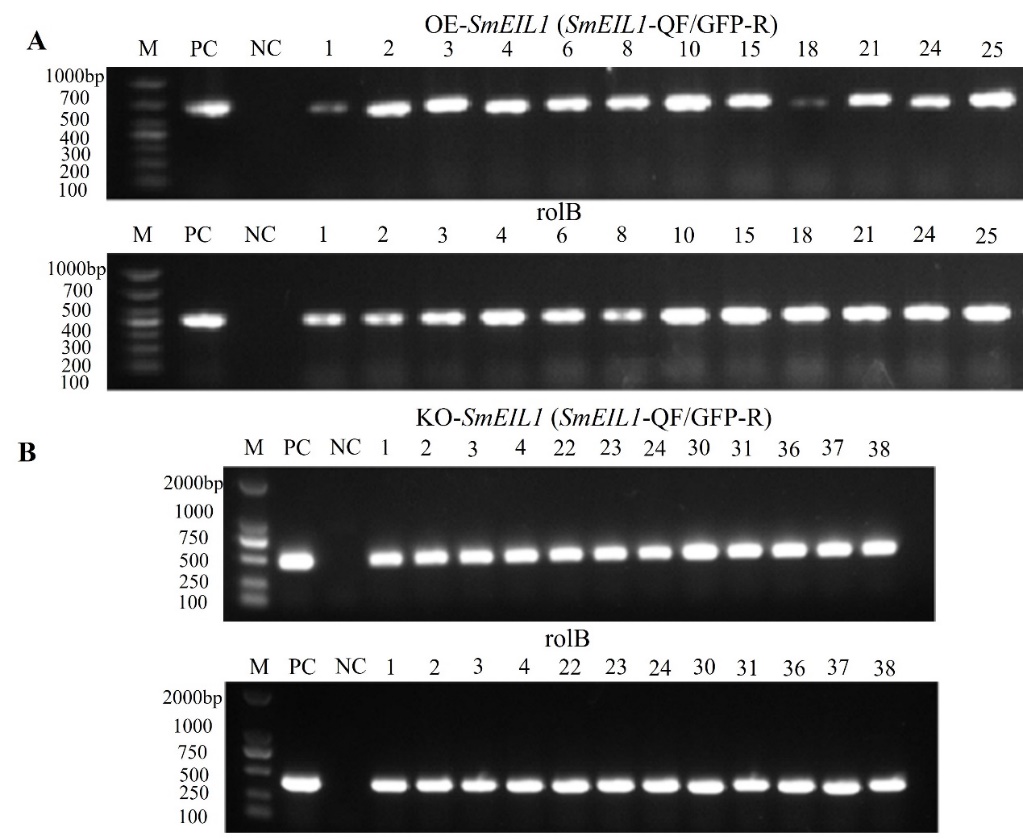


**Supplementary Figure 1.** Acquisition of transgenic hairy roots. **(A)** PCR identification of hair roots with *SmEIL1* overexpression. **(B)** PCR identification of hair roots knocked out by *SmEIL1*. PC: pHBSmEIL1-GFP and pCAMBIA1300-CRISPR/Cas9-SmEIL1 in C58C1 was used as positive control; NC: ddH_2_O was used as negative control.


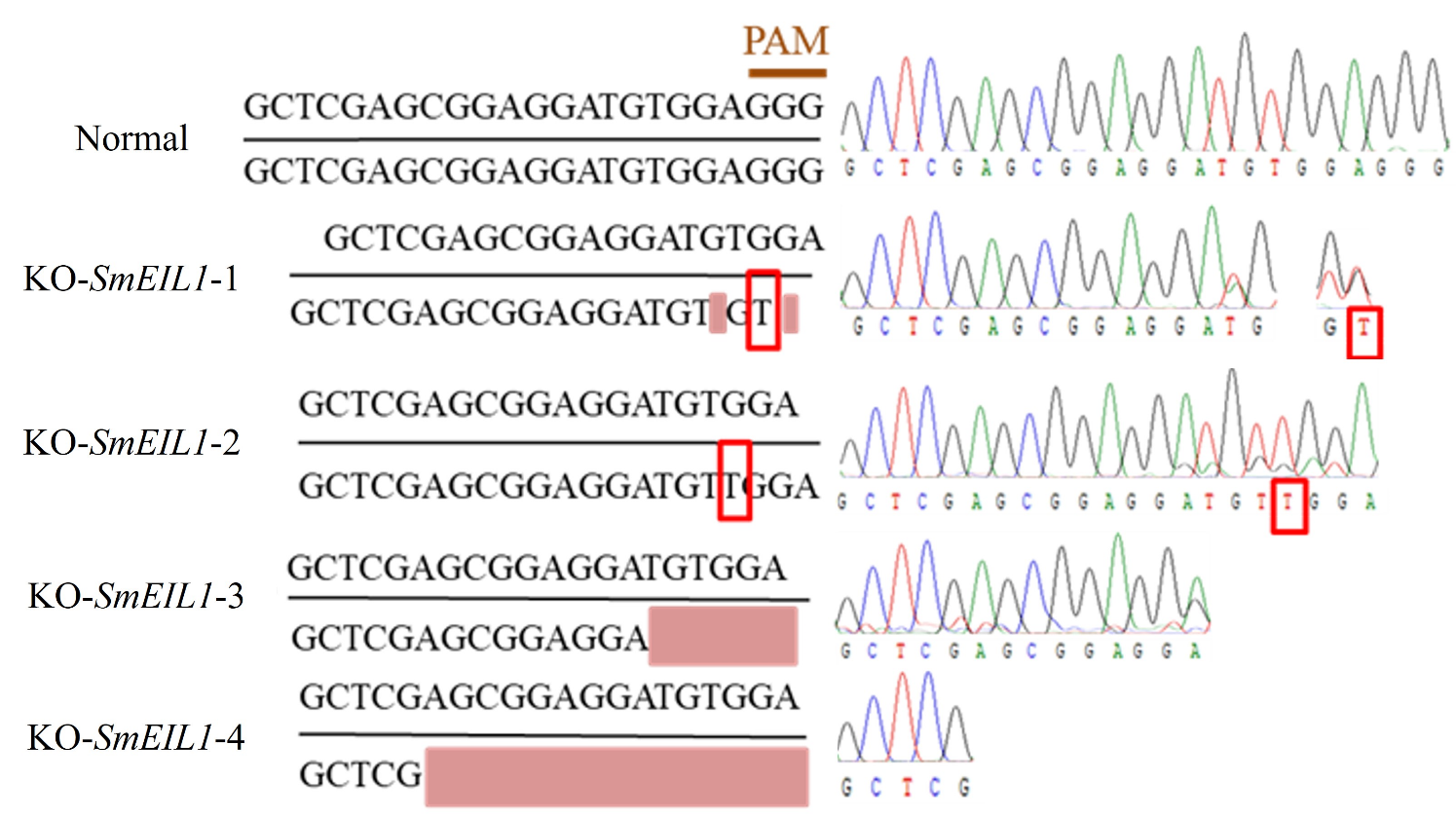


**Supplementary Figure 2.** Sequencing results of knockout hairy roots. Normal: Wild type sequence; PAM: NGG anchors the sequence.

**
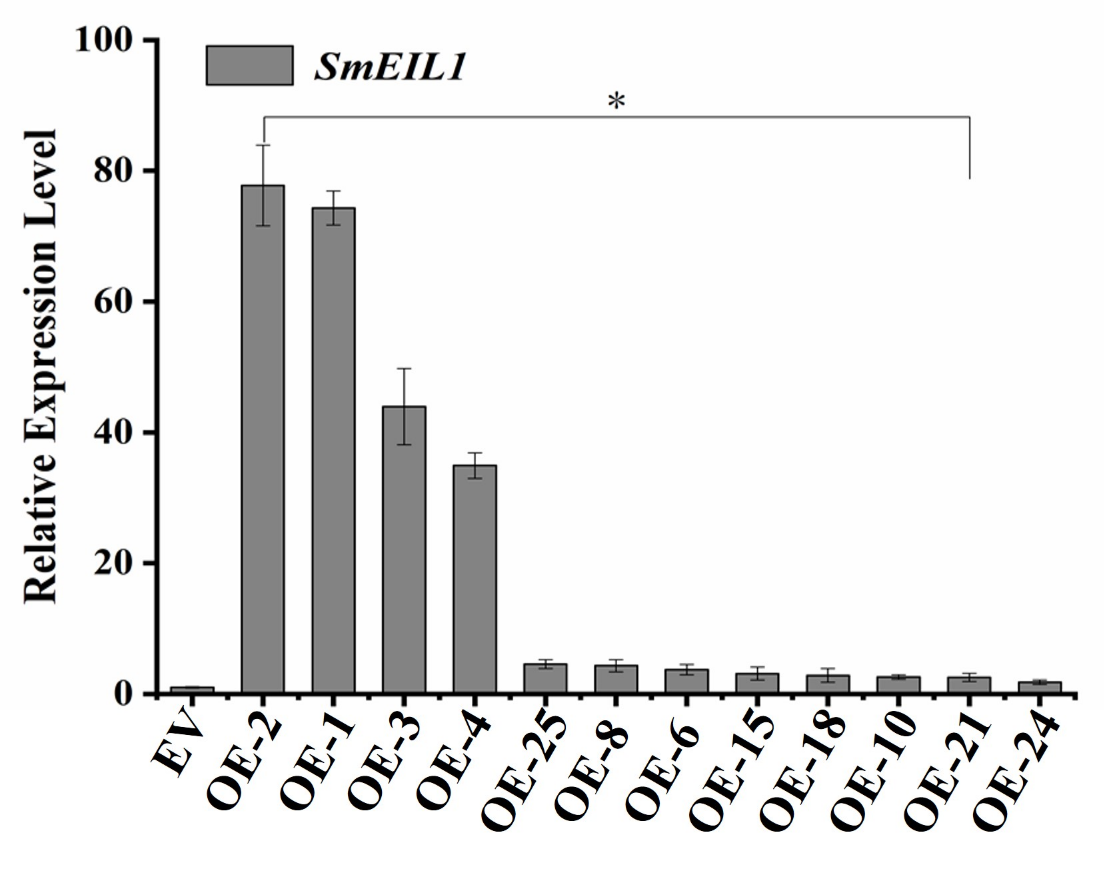
**

**Supplementary Figure 3.** Expression level of *SmEIL1* gene in overexpressed hairy roots. The

average expression level of the control group was set as 1; *SmActin* is an internal reference gene; The

error line represents the error for three biological replicates; The *t*-test was used for significant differences (**P*< 0.05).


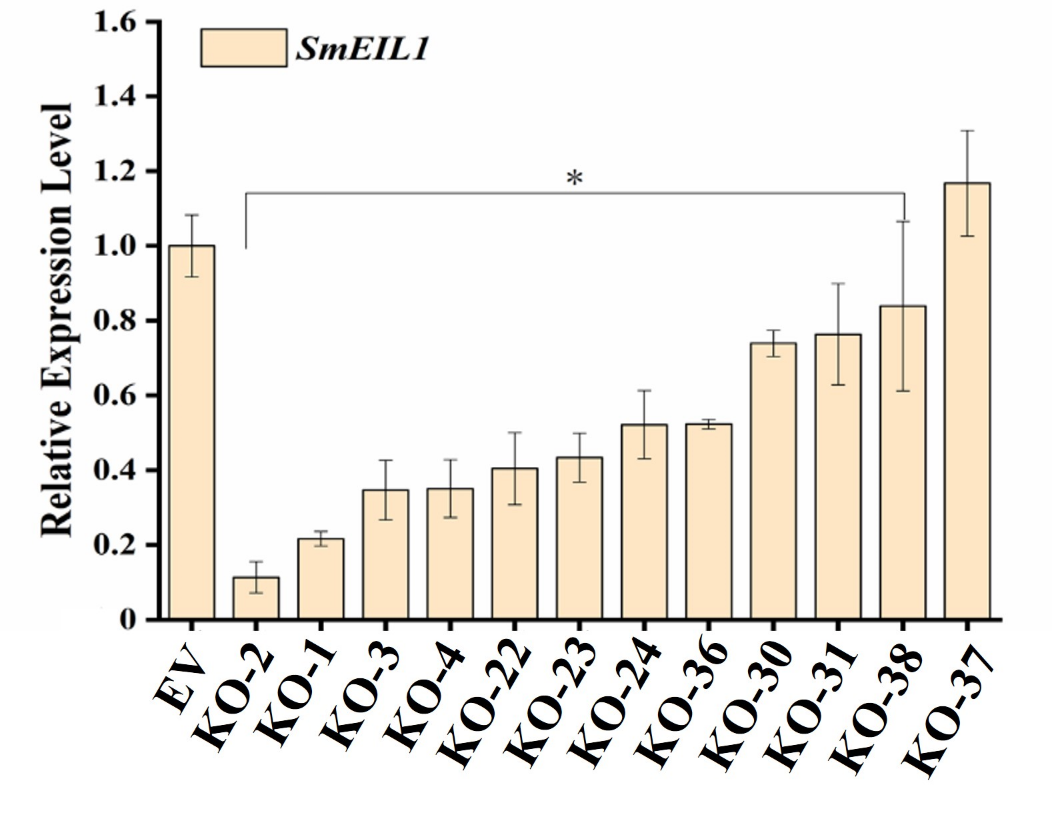


**Supplementary Figure 4.** The expression level of *SmEIL1* gene in hairy roots was knocked out. The

average expression level of the control group was set as 1; *SmActin* is an internal reference gene; The

error line represents the error for three biological replicates; The *t*-test was used for significant differences (**P*< 0.05).


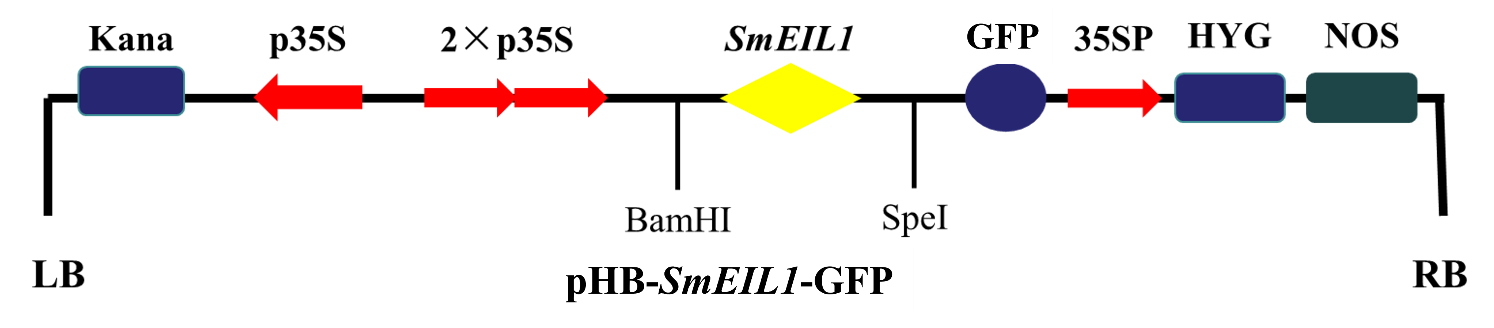


**Supplementary Figure 5.** pHB-SmEIL1-GFP vector.


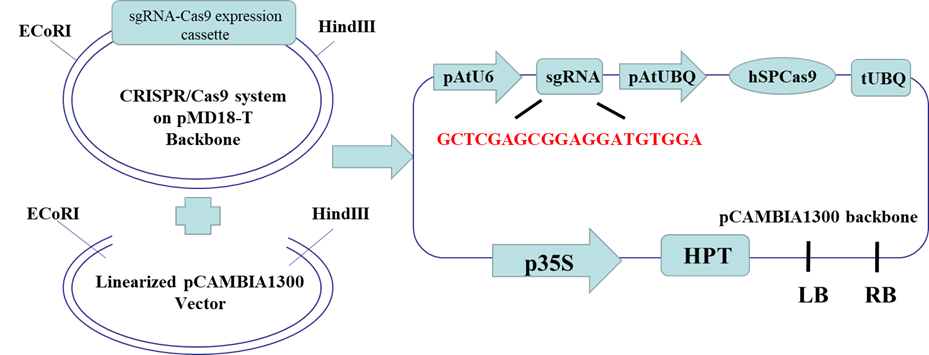


**Supplementary Figure 6.** pCAMBIA1300-CRISPR/Cas9-SmEIL1 gene knockout vector.

**Supplementary Table 1.** The primers information.

| Primers | Sequence (5’-3’) |
| --- | --- |
| SmActin-QF | AGCACCGAGCAGCATGAAGATT |
| SmActin-QR | AGCAAAGCAGCGAACGAAGAGT |
| SmEIL1-QF | CTCAATCATTTGTCCAGCAGAAGC |
| SmEIL1-QR | CGGGTTGTGATGAATGTTGCTGTT |
| SmEIL1-KF | ATGATGATGTTCGAGGATATGGG |
| SmEIL1-KR | TTAGTACCACATTGCAACATCCTG |
| pHB-*EIL1*-*Bam*HI-F | CGGATCCATGATGATGTTCGAGGATATGGG |
| pHB-EIL1-SpeI-R | GACTAGTGTACCACATTGCAACATCCTGC |
| Sm*EIL1*-sgRNA-F | GATTGCTCGAGCGGAGGATGTGGA |
| Sm*EIL1*-sgRNA-R | AAACTCCACATCCTCCGCTCGAGC |
| rol B-F | GCTCTTGCAGTGCTAGATTT |
| rol B-R | GAAGGTGCAAGCTACCTCTC |
| pHB-GFP-R | TGGTGCAGATGAACTTCAGGGT |
| M13F | CGCCAGGGTTTTCCCAGTCACGAC |
| CRISPR-*SmEIL1*-F | GGAACGCAGCATAATACAAGATCT |
| CRISPR-*SmEIL1*-R | TGAAGTACATGTTGAAGATGATGGA |
| SmPAL1-QF | GATAGCGGAGTGCAGGTCGTAC |
| SmPAL1-QR | CGAACTAGCAGATTGGCAGAGG |
| SmTAT1-QF | CAACTGCTGGTCTTCCACAAAC |
| SmTAT1-QR | GCGAGCCAAAACGGACA |
| SmC4H1-QF | CCAGGAGTCCAAATAACAGAGCCG |
| SmC4H1-QR | GCCACCAAGCGTTCACCAAGAT |
| SmHPPR1-QF | TGACTCCAGAAACAACCCACATT |
| SmHPPR1-QR | CCCAGACGACCCTCCACAAG |
| Sm4CL1-QF | ATTCGCATTCGCATTTCTCGG |
| Sm4CL1-QR | GCGGCGTAGTGCTTCACCTTT |
| SmRAS1-QF | CGAGATCGCCTACTCCAAGTTCAAG |
| SmRAS1-QR | AGATGGCGTTACCGAAGTATCCCTG |
| SmCYP98A14-QF | GGTCTGTACCGTCGTCCTCTTCTCC |
| SmCYP98A14-QR | ACAAGGCTGGTATTTGGGAAAAGGT |
| SmDXS2-QF | TTGGAGATTGGGAAGGGAAGGAT |
| SmDXS2-QR | AGGCTTGCAGAATCTCGCATCAG |
| SmDXR1-QF | CGACGAGAAAATCGGATACCTGG |
| SmDXR1-QR | CATACAAGAGCAGGACTCGAACCG |
| SmHMGR1-QF | TCGTTTTCAATAAGTCGAGTAGA |
| SmHMGR1-QR | ATTCTGAAGGAAGTCCAAAACAT |
| SmGGPPS1-QF | GCTGTGCTCGCAGGGGATG |
| SmGGPPS1-QR | ATCGCCGGTGCAGTTCAGG |
| SmCPS1-QF | GATCGCCTCGTCAATACCAT |
| SmCPS1-QR | TTCGAACCCACAAGTCATGT |
| SmKSL1-QF | GTGTGACCCTTCTGCTAGCA |
| SmKSL1-QR | TGCATTGTCTTGGGAAGATG |
| SmCYP76AH1-QF | TGCCCAACTTCGCCGACTACTTC |
| SmCYP76AH1-QR | GTGTTCGTGTCCGATCCTCCCAC |
| pB42AD-Sm*EIL1*-*Eco*RI-F | GATTATGCCTCTCCCGAATTCATGATGATGTTCGAGGATATGGG |
| pB42AD-Sm*EIL1*-*Xho*I-R | AGAAGTCCAAAGCTTCTCGAGGTACCACATTGCAACATCCTGC |
| *SmCPS1*-EBS-*Eco*RI-F | AATTCCGGCATGAATCTAGCTCGGCATGAATCTAGCTCGGCATGAATCTAGCTC |
| SmCPS1-EBS-XhoI-R | TCGAGAGCTAGATTCATGCCGAGCTAGATTCATGCCGAGCTAGATTCATGCCGG |
| SmHMGR1-EBS-EcoRI-F | AATTCGGAATCCATGCAAACTGGAATCCATGCAAACTGGAATCCATGCAAACTC |
| SmHMGR1-EBS-XhoI-R | TCGAGAGTTTGCATGGATTCCAGTTTGCATGGATTCCAGTTTGCATGGATTCCG |
| pGBKT7-Sm*EIL1*-*Eco*RI-F1 | ATGGCCATGGAGGCCGAATTCATGATGATGTTCGAGGATATGGG |
| pGBKT7-Sm*EIL1*-*Sai*I-R1 | ATGCGGCCGCTGCAGGTCGACCGCCTGCTCCTGCGACTG |
| pGBKT7-Sm*EIL1*-*Eco*RI-F2 | ATGGCCATGGAGGCCGAATTCAGGAGGAAGAAGATGTCGAGGG |
| pGBKT7-Sm*EIL1*-*Sai*I-R2 | ATGCGGCCGCTGCAGGTCGACTTCCTCCTGGTTGATGATGGC |
| pGBKT7-Sm*EIL1*-*Eco*RI-F3 | ATGGCCATGGAGGCCGAATTCGCCCTGGCCCGGGAG |
| pGBKT7-Sm*EIL1*-*Sai*I-R3 | ATGCGGCCGCTGCAGGTCGACTTAGTACCACATTGCAACATCCTG |
| pXY104-*SmEIL1*-*Bam*HI-F | CGGGATCCATGATGATGTTCGAGGATATGGG |
| pXY104-*SmEIL1*-*Sai*I-R | GCGTCGACGTACCACATTGCAACATCCTGC |
